# Unsaturated Fatty Acid, *cis*-2-Decenoic Acid, in Combination with Disinfectants or Antibiotics Removes Pre-Established Biofilms Formed by Food-Related Bacteria

**DOI:** 10.1371/journal.pone.0101677

**Published:** 2014-07-07

**Authors:** Shayesteh Sepehr, Azadeh Rahmani-Badi, Hamta Babaie-Naiej, Mohammad Reza Soudi

**Affiliations:** Department of Biology, Alzahra University, Tehran, Iran; University of Birmingham, United Kingdom

## Abstract

Biofilm formation by food-related bacteria and food-related pathogenesis are significant problems in the food industry. Even though much disinfection and mechanical procedure exist for removal of biofilms, they may fail to eliminate pre-established biofilms. *cis*-2 decenoic acid (CDA), an unsaturated fatty acid messenger produced by *Pseudomonas aeruginosa*, is reportedly capable of inducing the dispersion of established biofilms by multiple types of microorganisms. However, whether CDA has potential to boost the actions of certain antimicrobials is unknown. Here, the activity of CDA as an inducer of pre-established biofilms dispersal, formed by four main food pathogens; *Staphylococcus aureus*, *Bacillus cereus, Salmonella enterica* and *E. coli*, was measured using both semi-batch and continuous cultures bioassays. To assess the ability of CDA combined biocides treatments to remove pre-established biofilms formed on stainless steel discs, CFU counts were performed for both treated and untreated cultures. Eradication of the biofilms by CDA combined antibiotics was evaluated using crystal violet staining. The effect of CDA combined treatments (antibiotics and disinfectants) on biofilm surface area and bacteria viability was evaluated using fluorescence microscopy, digital image analysis and LIVE/DEAD staining. MICs were also determined to assess the probable inhibitory effects of CDA combined treatments on the growth of tested microorganisms' planktonic cells. Treatment of pre-established biofilms with only 310 nM CDA resulted in at least two-fold increase in the number of planktonic cells in all cultures. While antibiotics or disinfectants alone exerted a trivial effect on CFU counts and percentage of surface area covered by the biofilms, combinational treatments with both 310 nM CDA and antibiotics or disinfectants led to approximate 80% reduction in biofilm biomass. These data suggests that combined treatments with CDA would pave the way toward developing new strategies to control biofilms with widespread applications in industry as well as medicine.

## Introduction

The biofilm mode of growth is a basic survival strategy deployed by microorganisms in a wide range of environmental, industrial and clinical settings [1]. Biofilms are defined as sessile communities of cells attached to each other and/or to surfaces or interfaces which are embedded in a self-produced matrix of extracellular polymeric substances (EPS) [2], [3]. A function frequently attributed to EPS is their general protective effect on sessile microorganisms against adverse conditions including presence of most antimicrobial agents [3]. This is supposed to be due mainly to physiological characteristics of biofilm bacteria, but also to a barrier function of EPS [4]. According to Körstgens *et al.* [5] the EPS matrix also provides biofilm mechanical stability by filling and forming the space between the bacterial cells, keeping them together.

Biofilm formation by food-related bacteria and food-related pathogenesis are significant problems in the food industry. The attachment of the bacteria to the food product or the product contact surfaces leads to serious hygienic problems and economic losses due to food spoilage [6], [7].

For the sanitation and removal of biofilms in food industry, chemical agents and mechanical forces (sonication, flushing, etc.) are parameters often involved simultaneously. Mechanical actions only allow the removal of the biofilms from the surfaces and once established, biofilms are harder to be removed completely [8]. They also cannot kill biofilms and biofilm cells might later re-attach to other surfaces and form a biofilm [8], [9]. Thus, disinfection procedure is indispensible with the intention of killing them. However, it is important to note that most of the disinfection processes that are implemented are based upon the results of planktonic tests [10]. Therefore, such tests do not mimic the behavior of sessile cells and can be highly ineffective when applied to control biofilms. Biofilms have been reported as possessing susceptibilities towards antimicrobials that are 100–1000 times less than equivalent populations of planktonic counterparts [11]. If a microbial population faces high concentrations of an antimicrobial product, susceptible cells will be inactivated. Although some cells may possess a degree of natural resistance and physiological plasticity or they may acquire it later through mutation or genetic exchange. These processes allow the microorganisms to survive and grow [4].

To address the need for novel and improved measures against biofilms especially pre-established biofilms, a clear strategy is to study the biofilm life cycle and identify key trigger points that regulate biofilm development. To control biofilm, the last stage of biofilm development presents several advantages, where a coordinated dispersal of biofilm cells is possible. Induction of biofilm dispersal could potentially use the microorganisms' own energy to remove established biofilms, revert cells to a planktonic phenotype and restore their susceptibility to disinfectants and antibiotics.

It has been recently reported that *P. aeruginosa* produces an un-saturated fatty acid, *cis*-2-decenoic acid (C_10_: Δ^2^, CDA), which is capable of inducing the dispersion of pre-established biofilms by multiple types of bacteria [12]. Furthermore, CDA is also capable of inducing dispersion in biofilms of *Candida albicans*, indicating that this signalling molecule is involved in inter-species and inter-kingdom signalling where it can modulate the behavior of other microorganisms that do not produce the signal [12]. CDA is a promising candidate for control of biofilms in different industrial and clinical settings as it has a broad-spectrum of activity in addition to the fact that it has no cytotoxic effects to human cells at nano-molar ranges [13]. However, whether CDA has potential to boost the actions of certain disinfectants and antibiotics is unknown.

Therefore, in the current work, the ability of nano-molar concentrations of CDA to induce dispersal in pre-established biofilms, formed by four main food-borne biofilm producer bacteria (*Bacillus cereus*, *Staphylococcus aureus*, *Salmonella enterica* and *E. coli*) as well as to remove and kill their biofilms when combined with biocides or antibiotics were studied? Besides, the ability of CDA to increase the inhibitory effects of antimicrobials on the growth of tested microorganisms' planktonic cells was investigated.

## Materials and Methods

### Bacterial strains, media and growth conditions

The microorganisms used in the present study included *E. coli* (ATCC 25922), *Staphylococcus aureus* (ATCC 25923), *Bacillus cereus* (ATCC 11778) and *Salmonella enterica* (ATCC 14028). Overnight cultures were grown at optimum temperature for each microorganism in Luria Bertani (LB) medium (Merck, Germany) for *E. coli, B. cereus* and *S. enterica* and in Tryptic Soy Broth (TSB) medium (Merck, Germany) for *S. aureus*. Biofilm experiments were performed in 1/5 strength LB for *E. coli*, *B. cereus* and *S. enterica*, and in 1/5 strength TSB for *S. aureus*.

### Chemicals and antimicrobial compounds

Three different concentrations of CDA (U-Chemo, China) (100, 310 or 620 nM) were used. These concentrations were previously observed to have the most effect on inducing the dispersion of pre-established biofilms [12] with no cytotoxic effects on human cells [13]. Ethanol (10%) (Merck, Germany) was used as a carrier for CDA. Two commercial disinfectants, Epimax S (Epimax, Iran) and Percidine (Behban chemistry, Iran), were used for their widespread applications in food industry in Iran. Their active ingredients were hydrogen peroxide (45–50%) and peracetic acid (15%), respectively. Final concentration of 120 ppm hydrogen peroxide for Epimax S and 70 ppm peracetic acid for Percidine was used. These concentrations were respectively 3 and 4 times lower than the manufacturer's recommended concentration for disinfection purposes. This study also examined three antibiotics commonly used in medical and veterinary practice; ciprofloxacin (Sigma, USA) for both gram positive and gram negative tested microorganisms, vancomycin (Sigma, USA) for only gram positive bacteria, and ampicillin (Sigma, USA) for gram negative strains. Ciprofloxacin (Sigma) was used at a final concentration of 1 µg.ml^−1^, vancomycin at (4 µg.ml^−1^ and 256 µg.ml^−1^ for *S. aureus* and *B. cereus*, respectively) and ampicillin at 256 µg.ml^−1^. The concentrations of antibiotic selected for use were established in our laboratory to be effective against planktonic cells but have no inhibitory effect on the tested pathogens' biofilm cells.

### Biofilm dispersal bioassays in petri dishes

Biofilms were grown on the inside surface of petri dishes by using a semi-batch culture method in which the medium was replaced every 24 h. This was done to reduce the accumulation of native dispersion inducing factors and to allow mature biofilms form. Biofilms grown in this manner were then treated with three different concentrations of CDA (100, 310 or 620 nM) as dispersion inducer or just the carrier (10% ethanol) as a control to release cells into the bulk liquid and evaluate dispersed cell number by measuring the optical density (OD). To cultivate biofilms, overnight cultures of tested microorganisms were diluted 1∶1,000 into fifteen ml of growth medium, (except for *B. cereus* that was diluted 200 times), inoculated in sterile petri dishes and incubated at room temperature with 30 rpm shaking. Medium in the plates was replaced every 24 h for 5 days. After the last exchange of medium, the cells were allowed to grow for about 1 h and then dispersion induction was tested by replacing the growth medium with fresh medium containing one of the indicated concentrations of CDA or just the carrier as a control and the cells were incubated for a further 1 h. Afterward, Medium containing dispersed cells was transferred by pipette to a 50 ml Erlenmeyer and was homogenized for 30 s at 5,000 rpm with a WiseTis-Homogenizer model HG-150 (Daihan Scientific Co., Ltd., Korea) to ensure the separation of cells. The cell density was then determined based on the OD_600_ with an UV/VIS spectrophotometer model T80^+^ (PG Instruments, Ltd., China). Biofilm dispersal bioassays were performed in triplicates in at least three individual experiments for each concentration.

### Dispersion bioassays of biofilms in biofilm tube reactors

Biofilms were also grown on the interior surfaces of tubing reactors. A continuous once-through tube reactor system was configured by using eight silicone reactor tubes (40-cm length by 3-mm inner diameter), connected to an eight-roller head peristaltic pump (Baoding Longer Precision Pump Co., Ltd., China) and medium reservoir, via an additional silicone tubing. Medium was pumped through the tubing to a closed effluent medium reservoir. The entire system was closed to the outside environment but maintained in equilibrium with atmospheric pressure by a 0.2-µm-pore-size gas-permeable filter fitted to medium reservoir. The assembled system was sterilized by autoclaving prior to inoculation. The silicone tubes were inoculated by syringe injection through a septum 1 cm upstream from each reactor tube, with 3 ml of overnight cultures of each microorganism. Bacteria cells were allowed to attach (static incubation) to the tubing for 1 h, after which the flow was started at an elution rate of 280 µl.min^−1^. After 5 days of biofilm cultures, the influent medium was switched from fresh medium in the test lines to one of the three concentrations of CDA. Control lines were switched to new lines containing just the carrier (ethanol 10%). Samples were collected in test tubes on ice and were subsequently homogenized and cell density was determined as mentioned above. All experiments were repeated three times.

The concentration of CDA that induced the most dispersal in the examined biofilms in both petri dish and tube reactor cultures was used for further studies.

### Combined CDA and biocide treatment of pre-established biofilms, formed on stainless steel discs

For disinfectants alone and combined CDA susceptibility testing, biofilms were formed on stainless steel (SS) type 316 discs with a surface area of 2.7 cm^2^, placed at the bottom of wells in 24-well plates. To grow biofilms, 2.5 ml of overnight cultures of each microorganism, previously diluted 1∶1,000 in biofilm medium (except for *B. cereus* as indicated above), was added to each well and incubated at room temperature with gentle shaking. Medium in the wells was replaced every 24 h for 5 days to allow mature biofilms form. Biofilms were then treated for 1 h with indicated concentrations of disinfectants alone or combined with 310 nM CDA as CDA at this concentration induced the most dispersal in the tested biofilms in both petri dish and tube reactor cultures. At the end of the experimental period, the SS discs were washed with PBS to remove non-adherent bacteria, carefully transferred to sterile glass tubes containing 1 ml of sterile 0.89% NaCl and washed with another 1 ml of 0.89% NaCl. To remove the biofilm from the SS discs, the glass tubes with the biofilms were placed in an ultrasonic bath for 10 min at room temperature. CFU were enumerated after plating on LB agar to assess bacterial viability. All experiments were repeated three times.

### Antibiotics combined CDA biofilm microtiter plate assays

To assess the effect of antibiotics alone and in combination with CDA, biofilms were grown on the inside surface of sterile polystyrene 96-well plates. For biofilm cultures, plates were inoculated with 150 µl/well of overnight culture containing the tested organism, previously diluted in growth medium (as indicated above) and incubated at 37 °C with shaking at 120 rpm. Medium within each well was replaced every 24 h for 5 days. Biofilms were then treated for 1 h with indicated concentrations of antibiotics alone or combined with 310 nM CDA. The plates were gently rinsed twice with PBS to remove planktonic and loosely adherent organisms. After rinsing, the plates were shaken dry and each well of each plate stained with 160 µl of an aqueous 0.1% crystal violet solution in distilled water. After allowing the stain to adhere to the biofilms for 15 min, each plate was again rinsed with PBS until no more stain could be rinsed from the plate. Each plate was again shaken dry, inverted and allowed to dry thoroughly for 30 min. Finally, 170 µl of a 30% acetic acid solution was pipetted into each well to desorb the adhered stain back into solution. After allowing 30 min for the adhered stain to dissolve into the destaining solution, the biofilm in each well was quantified via absorbance at OD_590_ using a ELx808 Absorbance Microplate Reader (BioTek Instruments, Inc., Winooski, VT) [14]. All experiments were repeated at least three times.

### Combined CDA and antimicrobial treatment of planktonic cells

We have evaluated the probable inhibitory effects on the growth of tested microorganisms' planktonic cells by biocides or antibiotics alone and in combination with three different concentrations of CDA (100, 310 or 620 nM). The MICs were determined in triplicate in Mueller-Hinton broth by using microdilution assay with bacteria at a density of 10^5^ CFU/ml. Plates were incubated for 24 h at optimum temperature for each bacterium. The lowest concentration of antibiotics or biocides where there was no growth after 24 h was taken as the MIC [15], [16].

### Flow cell (continuous-culture) biofilm experiments; disinfectants and antibiotics sensitivity assays and surface area coverage

To observe the effect of CDA combined antimicrobial treatments on biofilm surface area and bacteria viability, biofilms were also grown in continuous culture flow cells (channel dimensions, 1×4×40 mm). Appropriate sterile biofilm medium was pumped from a 5-Liter vessel through silicone tubing to the flow cell using an eight-roller-head peristaltic pump (Baoding Longer Precision Pump Co., Ltd., China) at a flow rate of 280 µl.min^−1^. Medium leaving the flow cell was discharged to an effluent reservoir via silicone tubing. The entire system was closed to the outside environment but maintained in equilibrium with atmospheric pressure by a 0.2-µm-pore-size gas-permeable filter fitted to each vessel. Channels were inoculated with overnight cultures of tested organism and incubated without flow for 1 h, at room temperature. After 48 h of biofilm cultures, the influent medium was switched from fresh medium in the test lines to the antimicrobials in combination with 310 nM CDA. Control lines were switched to new lines containing only examined antimicrobial agents. After 1 h treatment, biofilms were stained with a LIVE/DEAD *Bac*Light bacterial viability kit (Molecular Probes). The two stock solutions of the stain (SYTO 9 and propidium iodide) were diluted to 3 µl.ml^−1^ in biofilm medium and injected into the flow channels. Live SYTO 9-stained cells and dead propidium iodide-stained cells were visualized using epifluorescence microscopy (CETI, Belgium). 15 selected fields of view per flow cell were imaged in the XY plane, at regular intervals and across the entire channels. Image analysis (ImageJ Software, NIH) was performed and results were presented as the percentage of total biofilm surface reduction in cultures treated with combined CDA and antimicrobial treatments relative to the total biofilm surface in control cultures that were not exposed to CDA. Three replicates per experiment were used and at least 2 independent repetitions of experiments were performed.

### Statistical Analysis

All data were analyzed using analysis of variance (ANOVA) by the general linear model procedure of Minitab data analysis software (release 16, Minitab Inc., PA. USA). Pairwise comparisons were then made between all of the groups using Tukey's method. *P* values <0.05 were regarded as significant. All measurements were carried out in triplicate.

## Results

### Very low concentrations of CDA induce biofilm dispersal

**Figure 1:**
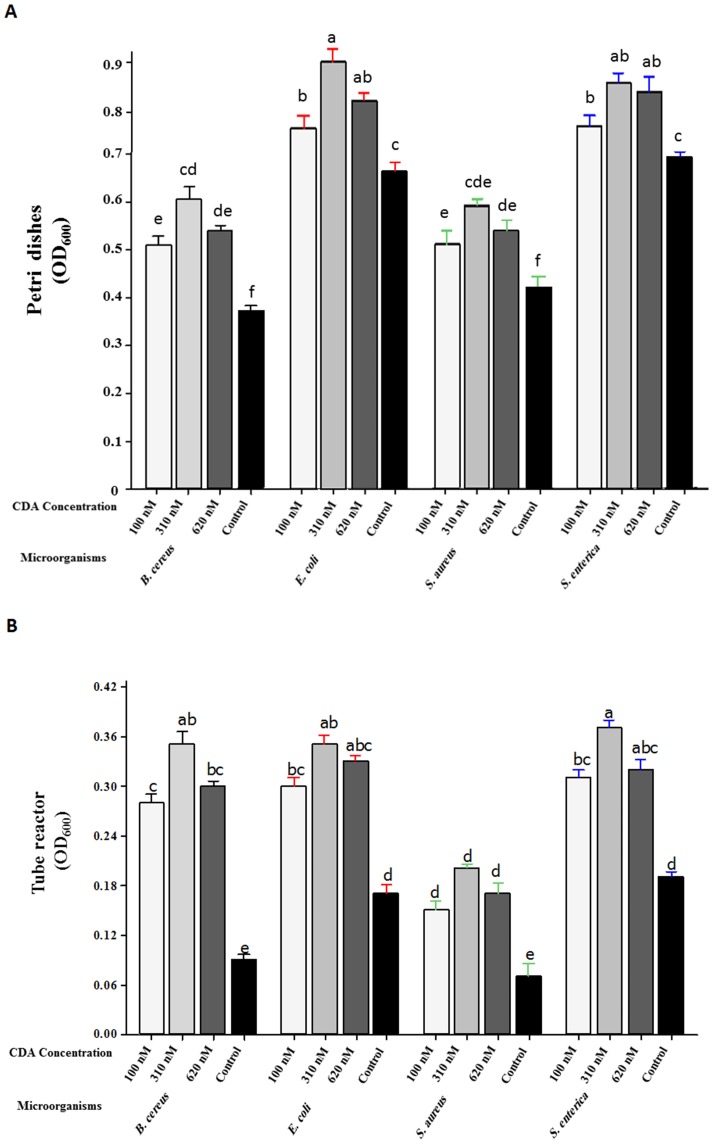
Induction of planktonic mode of growth in pre-established biofilms formed by food pathogens using CDA. (A) Biofilms were grown for 5 days in petri dishes in which the medium was replaced every 24 h. Dispersion induction was tested by replacing the growth medium with fresh medium containing three different concentrations of CDA (100, 310 or 620 nM) or just the carrier as a control and the cells were incubated for a further 1 h. Medium containing dispersed cells was then homogenized and cell density was determined by measuring the optical density. (B) After 5 days of biofilm growth in flow cell continuous cultures, the influent medium was switched from fresh medium in the test lines to three indicated concentrations of CDA and control lines were switched to new lines containing just the carrier. Effluent runoffs were then collected and cell density was determined by measuring the OD. Error bars indicate standard errors (n = 3) and mean values sharing at least one common lowercase letter shown above the bars are not significantly different (*P-value* <0.05).

We investigated the effect of exposure to nano-molar concentrations of CDA on pre-established biofilms in the petri dish cultures. In all cultures tested, CDA treatments resulted in a significant increase in the populations of planktonic cells released into the bulk liquid compared to untreated control samples (Figure 1A). The greatest effect was repeatedly observed with 310 nM CDA with at least two-fold increase in the number of planktonic cells. However, no significant differences were detected in the number of planktonic cells after exposure of *S. enterica* biofilms to 310 and 620 nM CDA (*P-value* <0.05) (Figure 1A). Following exposure to 310 nM CDA, the most significant increase in planktonic population was observed in the case of *E. coli* biofilms (OD_600_ = 0.9±0.02, SE, *P-value* <0.05) versus untreated controls (OD_600_ = 0.66±0.01, SE, *P-value* <0.05) (Figure 1A). The results from these experiments are summarized in Figure 1A. We also examined the effect of exposure to very low concentrations of CDA on pre-established biofilms grown in continuous cultures on the inner surface of silicone tubing. We again observed an increase in population of planktonic cells after treatment with CDA, indicating the release of biofilm bacteria into the effluent of cultures treated with CDA. As for semi-batch biofilm cultures, the most increase in population of planktonic cells in the effluents, with more than two-fold increase in the number of planktonic cells in comparison with control biofilms were observed when cultures were treated with 310 nM CDA (Figure 1B).

At this concentration, the most significant increase in population of planktonic cells was observed in *S. enterica* biofilms (OD_600_ = 0.37±0.01, SE, *P-value* <0.05) compared to results for untreated controls (OD_600_ = 0.19±0.005, SE, *P-value* <0.05) and no significant differences were detected between *B. cereus* and *E. coli* biofilms.

The results from these two different dispersal bioassays demonstrated the ability of nano-molar ranges of CDA to stimulate the release of cells from pre-established biofilms formed by different species of food related- bacteria.

### Antimicrobial combined CDA survival assays of pre-established biofilms on stainless steel and polystyrene surfaces

**Figure 2:**
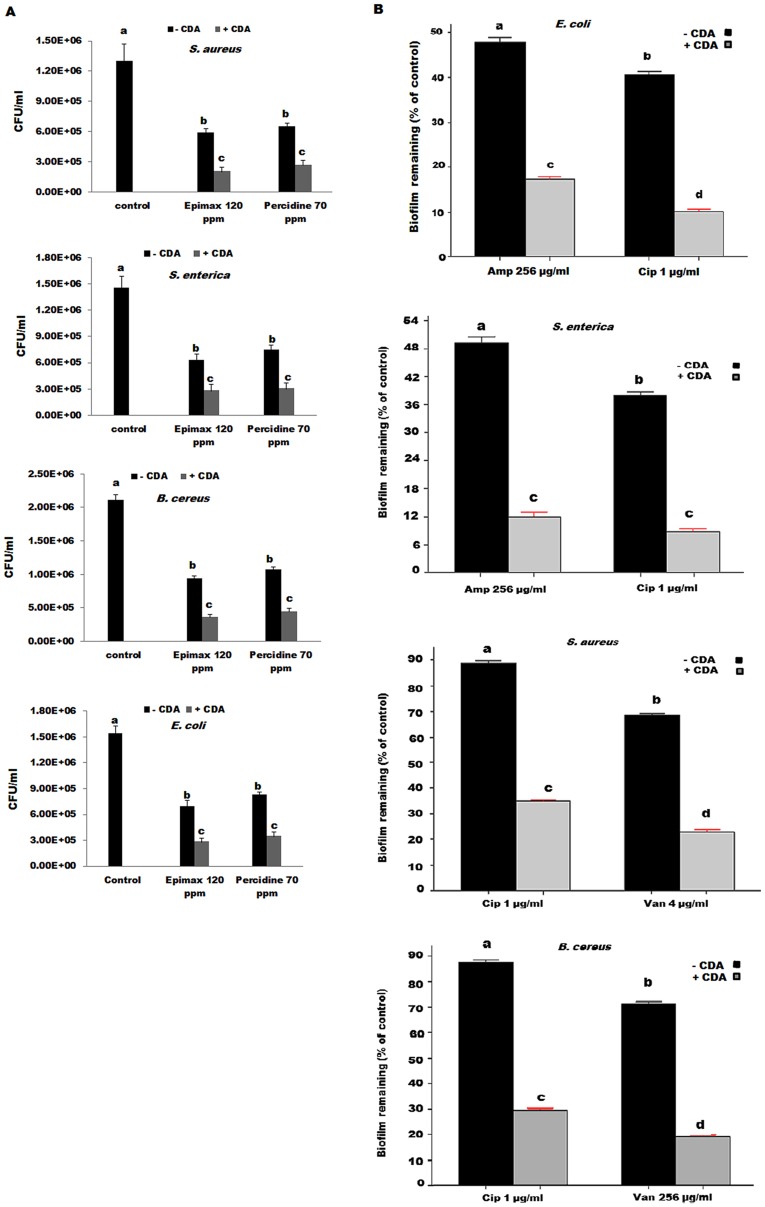
Effect of CDA combined antimicrobial treatments on eradication and killing of pre-established biofilms. (A) After 120 h of growth on the surface of SS discs, biofilms were treated for 1 h with biocides alone or combined with 310 nM CDA; CFU plate counts were then performed to assess the viability of the bacteria. (B) The amount of biofilm remaining was determined by the absorbance at 590 nm of crystal violet after staining the 120 h different biofilms in a microtiter plate assay after treatment with tested concentrations of antibiotics alone (- CDA) or in combination with 310 nM CDA (+CDA) for 1 h. All readings are corrected to reflect 0% and 100% controls (blank well, 0%; biofilms without any treatments, 100%). Error bars indicate standard errors (n = 3) and mean values sharing at least one common lowercase letter shown above the bars are not significantly different (*P-value* <0.05).

To examine the effect of CDA combined antimicrobial agents on removal of biofilms; we tested Epimax S (hydrogen peroxide) and Percidine (peracetic acid) against pre-established biofilms grown on the surface of SS discs, in the presence and absence of 310 nM CDA. When 120 h biofilms were treated in the absence of CDA, both disinfectants caused approximate two-fold decrease in CFU counts compared to the untreated controls, while combined exposure of cultures to 310 nM CDA and 70 ppm Percidine or 120 ppm Epimax S, resulted in approximate five-fold decrease in CFU counts. No significant differences were observed between these two different combinational treatments in reduction of CFU counts (*P-value* <0.05). The results from these experiments are illustrated in Figure 2A.

We have also tested effectiveness of CDA combined with three antibiotics (ciprofloxacin, vancomycin and ampicillin). We observed that combined treatments with both CDA and antibiotics had a significant effect on removing pre-established biofilms formed by examined microorganisms on polystyrene surfaces. For example, ciprofloxacin treatment of biofilms formed by *S. aureus* and *B. cereus* caused approximately 11% and 13% reductions in their biofilms, respectively (compared to biofilms without any treatments) while combined treatment of their biofilms with 1 µg of ciprofloxacin and 310 nM CDA resulted in 87% and 89% removal of their biofilms, respectively.

Significant differences were detected between two different combinational treatments applied for gram positive and gram negative bacteria; since the combination of CDA and ciprofloxacin was more effective than CDA combined ampicillin to eradicate biofilms formed by gram negative organisms. Similarly, combined treatments with both CDA and vancomycin were more effective to eliminate biofilms formed by gram positive bacteria. Results from these experiments are summarized in Figure 2B.

Thus, combined treatments using only low concentrations of CDA together with biocides or antibiotics were highly effective in removal and killing of pre-established biofilms formed by food pathogens.

### Combined CDA and antimicrobial treatment of planktonic cells

To further investigate the effect of CDA on the sensitivity of tested microorganisms towards antimicrobial agents, we also evaluated very low concentrations of CDA for any inhibitory effects on growth of their planktonic cells. Compared to antibiotics or biocides alone, combination of antimicrobial treatment with nano-molar concentrations of CDA had no additional inhibitory effects on the growth of planktonic cells; for that reason only Minimum Inhibitory Concentrations (MICs) for antibiotics and disinfectants alone are presented in Table 1.

**Table 1:** MICs of tested microorganisms' planktonic cells to examined disinfectants and antibiotics.

Bacteria	Epimax S (ppm)	Percidine (ppm)	Ampicillin (µg.ml^−1^)	Vancomycin (µg.ml^−1^)	Ciprofloxacin (µg.ml^−1^)
*E. coli*	20	10	128	-	0.125
*S. aureus*	10	10	-	1	0.25
*B. cereus*	10	10	-	64	0.125
*S. enterica*	20	10	128	-	0.25

### Biofilm surface coverage reduction by CDA combined biocides or antibiotics

**Figure 3:**
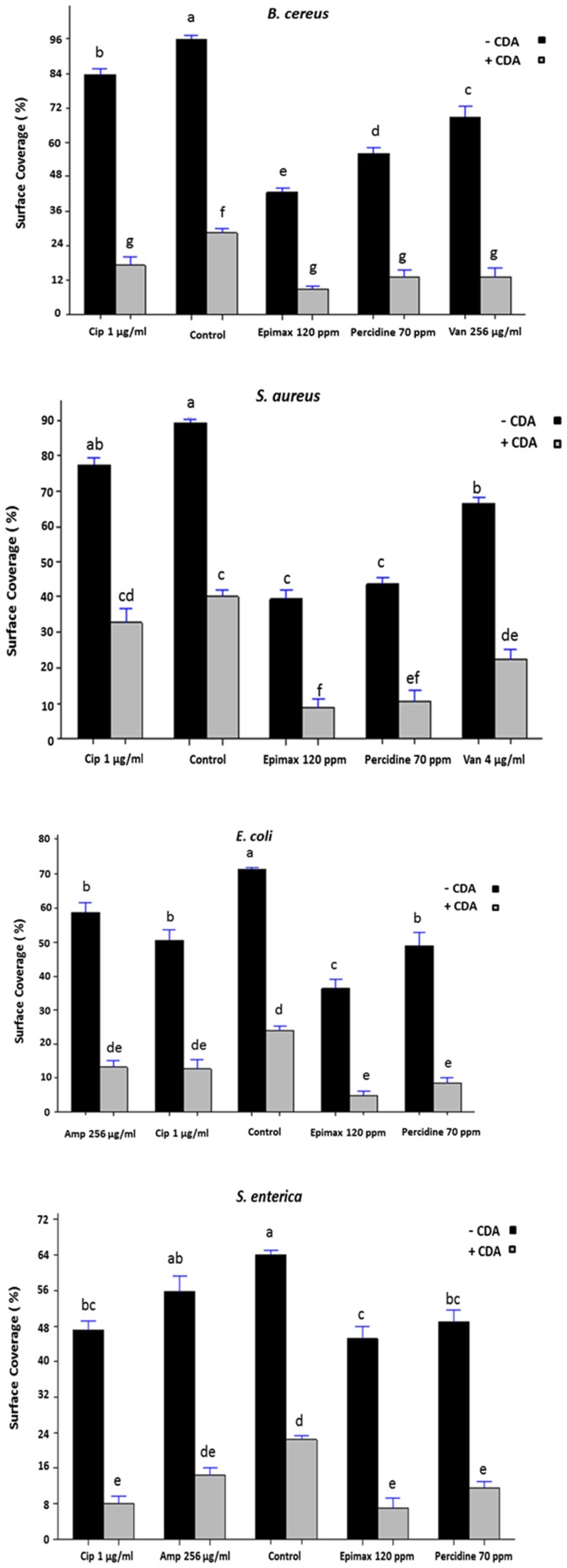
Effect of CDA combined disinfectant or antibiotic treatments on biofilms surface area. Following dispersion of biofilms by CDA, cells remaining on the surface are easily killed and removed by various disinfectants (Epimax S and Percidine) or antibiotics (vancomycin; Van, ampicillin; Amp, ciprofloxacin; Cip) in biofilms grown in continuous culture flow cells. Pre-established biofilms were grown for 48 h without any treatment and then were treated with indicated concentrations of antimicrobials alone (- CDA) or combined with 310 nM CDA (+ CDA) for 1 h, stained with LIVE/DEAD staining and quantified (percent surface coverage) using digital image analysis. The bars show the levels of biofilm biomass after treatment with antimicrobials alone or combined with 310 nM CDA. Error bars indicate standard errors (n = 3) and mean values sharing at least one common lowercase letter shown above the bars are not significantly different (*P-value* <0.05).

**Figure 4:**
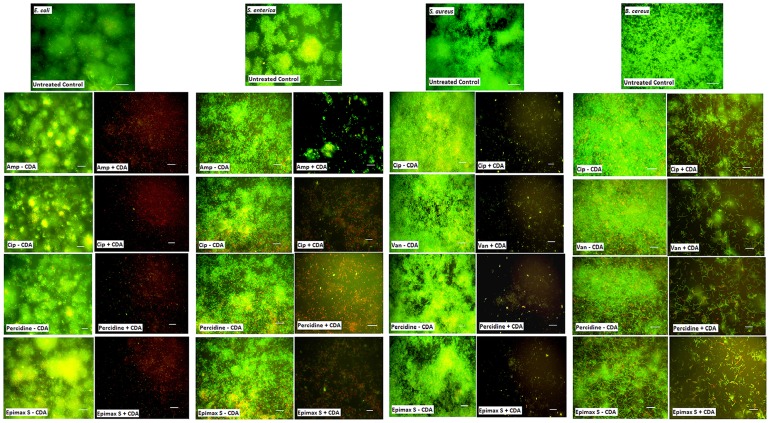
Effect of CDA combined antimicrobial treatments on killing of pre-established biofilms. CDA treatment reverses biofilm formation in pre-established biofilms and cells remaining on the surface are easily removed and killed various disinfectants (Epimax S and Percidine) or antibiotics (vancomycin; Van, ampicillin; Amp, ciprofloxacin; Cip) in biofilms grown in continuous culture flow cells. Pre-established biofilms were grown for 48 h without any treatment, then were treated with indicated concentrations of antimicrobials alone (- CDA) or combined with 310 nM CDA (+CDA) for 1 h and stained with LIVE/DEAD staining to allow analysis using fluorescence microscopy. The images show microscopic pictures of the biofilms on the surface of cover slip after combinatorial treatments. Images are top-down views (x-y plane); scale bars: 50 µm. Results are representative of 3 separate experiments.

To further examine the effect of CDA on biofilm surface area and bacteria viability, we also tested various disinfectants and antibiotics alone or combined with CDA against pre-established biofilms grown in continuous culture flow cells. When 48-h biofilms were treated in the absence of CDA, none of the disinfectants or antibiotics reduced biofilm biomass effectively (Figure 3). In contrast, after combined treatment, the biofilm cells remaining on the surface were easily removed and killed by antimicrobial compounds when examined by using the LIVE/DEAD staining kit (Figure 4). The most significant reduction in biofilm surface area (*P-value* <0.05) was observed when biofilms were treated with combination of Epimax S and 310 nM CDA. For example, this combination resulted in eradication of more than 90% of the *E. coli* biofilms from the surface (Figure 3).

Combined treatments with both CDA and antibiotics or biocides caused almost-complete eradication of pre-established biofilms.

## Discussion

The EPS matrix acts as a barrier in which diffusive transport prevails over convective transport [3]. EPS delay or prevent antimicrobials from reaching target microorganisms within the biofilm by diffusion limitation (like ciprofloxacin and ampicillin) [17], [18] and/or chemical interaction with the matrix material (like peroxides such as peracetic acid and hydrogen peroxide) [19]. Against such a drawback still oxidizing compounds (like peroxides) for their nonspecific mode of actions and because of variation in the chemical composition of biofilms are among widely used disinfectants in food industry in most countries including Iran. Several studies have shown that strategies to induce biofilm dispersal could potentially use the microorganisms' own energy to disrupt EPS and remove pre-established biofilms [20]. In a previous study Davies and Marques [12] showed that a synthesized signalling molecule by *P. aeruginosa* induces dispersion of pre-established biofilms in *P. aeruginosa* as well as many other strains of microorganisms. They concluded that CDA most likely induce the production of degradative enzymes of EPS by these microorganisms. Differential microarray analysis, by Rahmani *et al.* (under preparation) indicated that 100 nM CDA (added exogenously to *P. aeruginosa* pre-established biofilms) significantly up regulates the expression of *P. aeruginosa* genes including EPS, alginate, degradative enzyme (alginate lyiase; *algL*) and negative regulator for this EPS biosynthesis (*mucB*). Their results also showed that CDA down regulates the expression of genes involved in *P. aeruginosa* attachment to the surfaces (*cupA* and *cupB*), which results in reversion of biofilms to a population of planktonic cells with increased susceptibility to antimicrobial agents compared to their sessile counterparts (Rahmani *et al.*, under preparation). Therefore, in this investigation we first examined the action of nano-molar concentrations of CDA (as an inducer of biofilm dispersal) on dispersion of pre-established biofilms, formed by four main food-borne pathogenic or spoilage microorganisms. Our results interestingly showed that only 310 nM of the signal was enough to reverse pre-established biofilms, formed by distant genera of bacteria, to their planktonic mode of growths. Since disinfectants and antibiotics have greater bactericidal efficacy against planktonic bacteria than their sessile counterparts, the combination of CDA with common antimicrobial agents could have improved bactericidal efficacy. Thus, we then tried to remove and kill pre-established biofilms by using the combination of CDA and traditional disinfectants or antibiotics which are broadly used in food processing environments and their related medical issues, at concentrations that had no significant effects against biofilms, to reach a novel mechanism for enhancing the activity of these treatments through the disruption of biofilms. The results presented here demonstrated that following exposure to low concentrations of CDA, biofilm cells on the surface were easily detached and then killed by antimicrobial agents where the combination of 310 nM CDA with examined disinfectants (Percidine and Epimax S) or antibiotics (ciprofloxacin, vancomycin and ampicillin), when added to their solutions, resulted in approximate 80% reduction in biofilm biomass in all cultures.

Numerous strategies to control microbial biofilms have been proposed, with different degrees of success. In various industrial settings, a range of biocides and toxic metals (e.g., tin and copper) has been used for antifouling coatings and sanitizing purposes [21], [22]; however, these substances are not appropriate for use in food industries and clinical settings. In this work, we showed that CDA-based strategies to induce biofilm dispersal involve only nano-molar concentrations of CDA that should be safe to humans and to the environment. Besides, previous findings showed that CDA has no cytotoxic or stimulatory effect on human cells even at high concentrations (up to 250 µg.ml^−1^) [13]. Because CDA mediates the transition from a biofilm to a planktonic phenotype via a signalling mechanism (because acts at nano-molar concentrations which are consistent with all known cell-to-cell signalling molecules) rather than toxic effect, CDA-based biofilm control strategies would not be expected to select for resistant strains as seen with antibiotics. Therefore, in this study we examined two different combination of CDA; CDA combined disinfectants and CDA combined antibiotics, to introduce a promising strategy which is appropriate to control biofilms both in food industry and clinical settings.

While some free fatty acids have antimicrobial properties [23], [24] and play a vital role in maintaining the microbial flora of the skin [25], [26], we demonstrated that CDA does not inhibit bacterial growth at nano-molar ranges that induce biofilm dispersal. These results were highly in consistent with the results from Jennings *et al*. study [13] where they showed that CDA inhibited bacterial growth only at high (micro-molar to milli-molar) concentrations. This lack of growth inhibition at lower concentrations was not surprising since bacteria produce this unsaturated fatty acid and use it as a signalling molecule [12].

## Conclusions

Data from this study suggest that application of CDA prior to or in combination with disinfectants or antibiotics may allow for novel and improved strategies to control biofilms in industrial as well as clinical settings, with clear benefits such as reduced ecological impact and reduced treatment costs.
